# Tobacco Health Risk Awareness among Socially Disadvantaged People—A Crucial Tool for Smoking Cessation

**DOI:** 10.3390/ijerph15102244

**Published:** 2018-10-13

**Authors:** Marek Milcarz, Kinga Polanska, Leokadia Bak-Romaniszyn, Dorota Kaleta

**Affiliations:** 1 Department of Hygiene and Epidemiology, Medical University of Lodz, 90-647 Lodz, Poland; marek.milcarz@op.pl (M.M.); dkaleta@op.pl (D.K.); 2 Department of Nutrition in Digestive Tract Diseases, Medical University of Lodz, 90-647 Lodz, Poland; leokadia.bak-romaniszyn@umed.lodz.pl

**Keywords:** tobacco, active and passive smoking, health risk perception, tobacco-associated health risk awareness, socially disadvantaged populations, smoking cessation

## Abstract

The goal of this cross-sectional survey was to assess the level of knowledge on harmful effects of environmental tobacco smoke (ETS) exposure and active smoking among socially-disadvantaged people in Poland. The study was conducted among 1817 respondents aged 18–59 years, who used aid services from local social care institutions in Piotrkowski district. Majority of the participants were aware of the fact that smoking may cause serious diseases and lung cancer (92%). However, those percentages were lower for awareness of ETS and health risk (69.4%) and for awareness of smoking/ETS-associated risk of stroke and heart attack (57%, 68%). The male respondents and smokers had much higher odds of lacking knowledge that smoking causes serious diseases and lung cancer compared to the females (OR = 1.47 and OR = 1.86; *p* < 0.05) and non-smokers (OR = 2.35 and OR = 2.31; *p* < 0.001). In addition, those with temporary jobs and the unemployed had a higher risk of lack of knowledge on smoking and lung cancer risk (OR = 2.14 and OR = 1.66; *p* < 0.05) as well as ETS and the risk of stroke (OR = 1.52 and OR = 1.51; *p* < 0.05) as compared to those with permanent jobs. The smokers who were aware of four health consequences of smoking indicated an intention to quit smoking within the next month more frequently when compared to those who did not have the knowledge on all of the analyzed harmful effects of tobacco use (19.7% vs. 13.1%; *p* < 0.05). There is a need to improve knowledge on the dangers associated with active and passive smoking among socially disadvantaged populations.

## 1. Introduction

Socio-economic status (SES) is significantly associated with a smoking status and thus, with the smoking related morbidity and mortality [1]. Despite tobacco control measures, the rate of progress for smoking prevalence reduction has not been consistent across geographies/development status and within the countries between the groups with different SES. This indicates, as enunciated by the recent trends, that the drive to maintain the past rates of decline must not be considered obvious. Higher smoking prevalence and related health risks among socially disadvantaged groups are associated with an earlier smoking onset, heaviness of smoking and less successful quitting attempts as compared to the groups enjoying better life situations [2,3,4,5]. It needs to be stressed out that, unless action is taken to address the high prevalence of smoking among lower SES groups, we can expect a future widening of social inequalities in health.

From the tobacco control viewpoint, one of the major applicable strategies to drive down smoking rates and environmental tobacco smoke (ETS) exposure includes dissemination of the facts concerning harmful effects of tobacco—awareness of them is frequently associated with an intention to quit and intentions predict future quitting attempts in a consistent manner [6,7,8]. Awareness of harmful effects of tobacco smoke also contributes to preventing the non-smokers from taking up the habit [4,6]. In addition, it is important because understanding the threats that active and passive smoking pose helps to introduce smoking bans at homes [5]. Finally, if smokers are aware of the influence of their smoking on others, they might be encouraged to quit. 

What is important, the knowledge about harmful effects of active and passive smoking constitutes the first step to behavior modification, but it is not enough for quitting. A recent review of the perceived barriers to smoking cessation in selected vulnerable groups by Twyman et al. has described several groups of factors influencing cessation, covering individual and lifestyle issues (physical addiction, low confidence, behavioral habit, low motivation, failed past attempts, relaxation, stress and mood management, perceived mental health benefits, enjoyment, low health related knowledge), social and community barriers (lack of support from a health professional, socializing, lack of social support, high prevalence and acceptability of smoking in a community), living and working conditions (living and working circumstances, stressful situations, limited structure in a day to day life, boredom, social and geographical isolation, access to resources to quit), cultural, socio-economic and environmental factors (cultural norms, maintaining identity and socio-economic factors) [9].

Most existing studies have been focused on the impact of knowledge of harmful effects of smoking on intention to quit whereas the association between awareness/education and actual quit success has been less frequently investigated and is less clear. It also cannot be excluded that lack of awareness of health effects of smoking may be an active choice of an individual to justify his/her smoking. 

The existing studies indicate that gender, age, ethnicity, education, income and smoking status are associated with awareness of harmful effects of smoking [6,8,9,10,11,12,13,14,15,16]. In the developed countries, majority of people are aware of the association between smoking and general health, lung cancer and heart disease [6]. However, awareness of the association between smoking and other conditions such as stroke and the impact of ETS exposure on the health of non-smokers is less frequently reported. 

It needs to be emphasized that majority of the studies have been conducted among cigarette smokers (either from the general population or subgroups of people), whereas the studies among non-smokers are rarely conducted.

Given that having knowledge on the health effects of smoking is essential for behavior change, an examination of socio-economic differences in that knowledge can help to explain part of the pronounced SES differentials in smoking prevalence and cessation rates.

That was the rationale for selecting the study region (the Piotrkowski district within Lodzkie voivodship) and the study population (beneficiaries of government welfare assistance) for our analyses [17]. Piotrkowski district is a region where more than 90% of its residents are from rural areas. As evaluated by the United Nations Development Program (UNDP) the district took the 11th position among all 314 rural districts with the lowest indicators of social development in Poland. The Local Human Development Index (LHDI) (that includs Health, Education and Welfare Index) was 25.97 as compared to 39.28 for Lodzkie voivodship. In Poland the national and local institutions are responsible for administration of social aid. The right to the benefits provided under the system is given to individuals and families who are unable to cope with difficult life situations using their own empowerment, resources and abilities. In 2013, the proportion of individuals who received social aid to the overall population in Poland was 7.7%. In the same year, there were 91,618 residents living on the premises of Piotrkowski District among whom 11,867 people required support of social assistance institutions. More information about the district can be found in our previous publication [17].

This study intends to assess the level of knowledge on harmful effects of smoking and ETS exposure among the socially-disadvantaged populations in Poland, and to present the relevant correlates. Those data are crucial for effective tobacco control measures among this vulnerable population.

## 2. Material and Methods

### 2.1. Study Design and Population

Detailed characteristics of the methodology of the study have been published elsewhere [17,18,19]. Briefly, this cross-sectional survey was performed between October 2015 and February 2016 among people aged 18–59 years, who resided in Piotrkowski district and used aid services offered by the local welfare assistance institutions. For the purposes of this study, the poverty threshold as adopted by the social assistance institutions was used (income of no more than 634 PLN (148 Euro) for a single person monthly, and 514 PLN (120 Euro) for a family member monthly). Of 3636 individuals who met the inclusion criteria (including age and income limit) 1817 agreed in writing to participate in the study (a 49.97% response rate). Approval was obtained from the Bioethics Committee of the Medical University in Lodz (RNN/243/15/KE) and written informed consents were collected from all the study participants.

### 2.2. Questionnaire and Study Measures

Detailed information about the questionnaire and its contents is available in previous research papers [17,18,19]. The adopted research tool provided for the collection of information on the respondents’ socio-demographic status, including: age, gender, education, employment, subjective assessment of monthly income, subjective health state and declared health problems. In addition, data on tobacco smoking status and ETS exposure were collected. The current smokers category included: daily smokers (smoking one or more cigarettes per day over past 30 days) and the occasional smokers. The non-smoker category included never-smokers and ex-smokers. The total ETS exposure (number of hours of daily exposure to tobacco smoke at any place, including home, work and public areas) was categorized as: none, <1 h, 1–5 h, 5–8 h, and more than 8 h per day (for more details please see Milcarz et al. [17,18,19]. 

In an effort to assess the study population’s awareness of the negative health consequences of active smoking, the following questions were brought up: “Does smoking tobacco cause serious illnesses?”, “Does smoking tobacco cause stroke?”, “Does smoking tobacco cause heart attacks?”, “Does smoking tobacco cause lung cancer?”. The available answers included: “yes”, “no”, “don’t know”. Affirmative responses classified the respondents as “aware”, while negative and “don’t know” responses classified them as “unaware”.

Similarly, ETS-related health risks were investigated. The following questions were asked: “Does passive smoking cause serious illnesses?”, “Does passive smoking cause stroke?”, “Does passive smoking cause heart attacks?”, “Does passive smoking tobacco lung cancer?”. The available answers included: “yes”, “no”, “don’t know”. The respondents were categorized as “aware of the negative health consequences of ETS exposure” (for all affirmative responses), and “unaware of the negative health consequences of ETS exposure” (for all negative and “don’t know” responses).

### 2.3. Statistical Analysis

For the statistical analysis the STATISTICA Windows XP version 10.0 program (StatSoft Poland Inc., Tulsa, OK, USA) was used. Statistical associations of particular characteristics categories in the analyzed subgroups were assessed using an extended version of the Mantel-Haenszel chi-squared test. The logistic regression analyses were applied to identify the risk factors for the lack of awareness of tobacco-related harm (separately for smoking-associated health risks and for ETS exposure-associated health risks). Initially, crude coefficients—ORs of the impact of odds variables on the lack of awareness of the tobacco-associated health risks were calculated. The multivariate logistic regression analysis included all of the variables significantly associated with the lack of awareness on smoking or ETS health consequences in any of the considered univariate models (*p* ≤ 0.05). Finally, the Awareness Index (AI) (aware of 0–3 negative health consequences of smoking and aware of all 4 negative health consequences smoking) in relation to the number of cigarettes smoked per day, number of previous quitting attempts and intention to quit smoking within the next 30 days was calculated among the smokers. 

## 3. Results

### 3.1. Characteristics of the Study Participants and Their Awareness of Smoking/ETS Exposure-Associated Health Risks

The characteristics of the study sample have been also descried in details elsewhere [17]. Socio-demographic data and smoking status are presented in the Supplementary Materials—Table S1. Moreover, Table S1 displays distribution of knowledge on health effects of smoking and ETS. Majority of the participants were aware of the fact that smoking may cause serious diseases (92.4% of the study participants, including 93.9% of the females and 89.5% of the males). That percentage was lower for awareness of ETS exposure and health risk (69.4%). The findings also revealed that 92% of the respondents believed that smoking causes lung cancer (a lower percentage of the respondents indicated awareness of the association between ETS and lung cancer (86.9%)). However, awareness of smoking-associated risk of stroke and heart attack was particularly low, i.e., 56.9%, 68.5%, respectively (similar percentages of the participants were noted for ETS and stroke: 56.7% as well as for ETS and heart attack: 68.4%).

### 3.2. Correlates of Lack of Awareness of Smoking and ETS-Associated Health Risks

The results of the logistic regression analyses, analyzing factors correlated with lack of awareness of smoking-associated health risks, have been presented in Table 1. The odds ratios (OR) and the 95% confidence intervals (CI) for lack of knowledge that smoking causes serious diseases and lung cancer showed that the male respondents had much higher odds of lacking that knowledge compared to the females (OR = 1.47; 95% CI: 1.02–2.11; *p* < 0.05 and OR= 1.86; 95% CI: 1.29–2.68; *p* < 0.01, respectively). In addition, compared to the non-smokers, the people who smoke were more likely not to be aware of the fact that smoking causes serious diseases and lung cancer (OR = 2.35; 95% CI: 1.63–3.38; *p* < 0.001 and OR = 2.31; 95% CI: 1.60–3.32; *p* < 0.001 respectively). Moreover, those with temporary jobs and the unemployed ones had a higher risk of lack of knowledge related to smoking and lung cancer risk as compared to those with permanent jobs (OR = 2.14; 95% CI: 1.16–3.96; *p* < 0.01 and OR = 1.66; 95% CI: 1.07–2.59; *p* < 0.05, respectively). 

In the case of knowledge about the fact that breathing other people’s smoke in causes serious illnesses or heart attacks in non-smokers, the subjective assessment of monthly income as sufficient to cover basic needs only was associated with lower odds of lack of that awareness compared to the assessment of income as not sufficient to cover even the basic needs (OR = 0.71; 95% CI: 0.56–0.90; *p* < 0.01 and OR = 0.76; 95% CI: 0.6–0.97; *p* < 0.05 respectively) (Table 2). The participants with temporary jobs and the unemployed ones were less aware of the fact that passive smoking increases the risk of stroke as compared to those with permanent jobs (OR = 1.52; 95% CI: 1.06–2.18; *p* < 0.05 and OR = 1.51; 95% CI: 1.22–1.87; *p* < 0.01 respectively). Other variables in the analyses were not significantly associated with the lack of awareness of ETS-associated health risks.

The socially disadvantaged smokers who were aware of four health consequences of smoking (awareness index (AI) = 4) indicated an intention to quit smoking within the next month more frequently than those who did not have the knowledge on any of the analyzed harmful effects of tobacco use (AI < 4) (19.7% vs. 13.1%; *p* < 0.05) (Table 3).

## 4. Discussion

The current study indicated that majority of the socially disadvantaged respondents were aware of the fact that smoking may cause serious diseases and lung cancer. However, those percentages were lower for awareness of ETS and health risk as well as for awareness of smoking/ETS-associated risk of stroke and heart attack. After having used socio-demographic proxy measures of vulnerability, it was demonstrated that the following population groups are less knowledgeable of tobacco’s harmful effects: male, smokers, those with temporary jobs or the unemployed ones and those with the worst socio-economic status. The smokers who were aware of the four health consequences of smoking indicated an intention to quit smoking within the next month more frequently when compared to those who did not have the knowledge on all of the analyzed harmful effects of tobacco use. Our study underlined the urgent need to improve knowledge on the dangers of active and passive smoking among the socially disadvantaged populations. It provides useful data for policy makers to be considered while developing media and educational and interventional campaigns for specific population subgroups.

Similarly, as in our survey also in other studies the awareness of smoking tobacco being a cause of serious illnesses as well as the relationship between smoking tobacco and lung cancer was uniformly high [20]. Across all the countries, except for China, where the Global Adult Tobacco Survey (GATS) was conducted, more than 90% of the population indicated such knowledge [20]. In comparison, the awareness of the relationship of smoking tobacco and heart attack and stroke was much lower. 

What is interesting, data from the current study indicated, similar to those observed in GATS-Poland, percentages of adults being aware of active smoking and related serious illnesses (92.4% vs. 91.5%) and lung cancer (92.0% vs. 92.6%) [21]. However, much lower percentages were noted for the disadvantaged population (the current study) as compared to the representative (GATS-Poland) population for knowledge about ETS and related health risk (69.4% vs. 81.4%), active smoking and an increased risk of stroke (56.9% vs. 61.8%) and heart attack (68.5% vs. 79.9%). Thus, even bigger gaps in knowledge among this vulnerable group are indicated. As it was pointed out by Siahpush et al. of four countries (USA, UK, Australia, Canada), smokers in Canada represented the highest awareness of the smoking related risk, which authors explained by the strong anti-smoking education campaigns in Canada [1]. At the time that we have performed the survey, pictorial warnings were not present on cigarette packages. They were introduced by the Amendment of Law in 2016. Therefore, it would be interesting to evaluate if this action improved population’s knowledge and perception of harmful effects of smoking.

The existing studies indicate that knowledge on the harmful effects of cigarette smoking varies by socio-demographic factors including sex, age, ethnicity, education, income and smoking status [6]. As an example, in a Bangladesh survey it has been demonstrated that women, the illiterate and residents of urban slums were less knowledgeable of tobacco’s harmful effects [6]. In our study population, the men and smokers were less knowledgeable of tobacco’s harmful effects as compared to the women and non-smokers. Our results are in agreement with those obtained based on GATS-Poland (women and non-smokers had a slightly higher level of knowledge about dangers of smoking) [21]. Considering gender trends, in the analysis by Gupta and Kumar there was marginally higher awareness amongst males in China and India and slightly higher awareness amongst females in Russian Federation and Ukraine [20]. That demonstrates country specific variations in harmful perception by gender. Majority of the existing studies indicate that educational attainment and income are associated with knowledge on tobacco’s harmful effects [6,8,9,10,11,12,13,14,15,16]. It needs to be pointed out that our study was conducted among the people who used aid services from the local social care institutions so they mostly represented the sector with a lower educational level (only 5% of the participants had a university degree) and income (socio-economically disadvantaged persons are defined as those whose income threshold does not exceed $158 USD (634 PLN) per month for individuals, and $128 USD (514 PLN) per month for family members) [17,18,19]. That can explain the general lack of significant differences with that respect. 

Our assessments indicated that smokers who were aware of four health consequences of smoking expressed an intention to quit smoking within the next 30 days more frequently as compared to those who did not have the knowledge on all of the analyzed harmful effects of tobacco use. This is crucial information for the policy makers and institutions conducting anti-smoking interventions as by increasing a population’s knowledge and awareness of smoking attributable risk the willingness to quit smoking can be stimulated. It would be also of interest to look if there is an association between awareness of health consequences of smoking and actual quit success. 

It needs to be underlined that knowledge about harmful effects of active and passive smoking constitutes the first step to behavior modification, but it might be not enough for successful smoking cessation. In recent years much research has explored motivations and barriers to quitting smoking among specific disadvantaged groups including mental health patients, those living in socio-economically deprived areas and pregnant Aboriginal and Torres Strait Islander women. The following barriers to quitting smoking (in addition to the lack of knowledge) have been considered: physical addiction, poor self-efficacy, pro-smoking community norms and lack of willpower [9,22,23,24,25,26,27]. It is also hypothesized that financial stress and characteristics of smokers’ social environments influence smoking cessation [28,29]. In addition, disadvantaged populations may have difficulty in accessing healthcare, obtaining cessation advice, and fail to receive cessation treatment during a quitting attempt. Skepticism about treatment effectiveness might also contribute to the under-administration of treatment and lack of success [30].

The following strengths of the study need to be highlighted. For the first time, knowledge on harmful effects of smoking and ETS exposure has been verified among social care beneficiaries from a rural district in Poland. In addition, rather than focusing only on tobacco smokers (as it has been done in most of the studies), this survey assessed knowledge in all the groups of people as we have expected that inaccurate awareness of tobacco’s harmful effects influences users and non-users alike. In addition, we believe that awareness of ETS-related harms, which is less frequently investigated, is also crucial for effective tobacco control measures. Finally, the interviewer-administered questionnaires completed during the face-to-face interviews produced higher values of sensitivity and specificity than the self-administered questionnaires, and helped to reduce the non-response rate.

This study was also a subject to some limitations. Firstly, one limitation of the study is related to the cross-sectional study design. What is more, a possible limitation of an epidemiological study might be related to the high non-response rate. Nevertheless, the response rate in our study was 50%, which is comparable to the participation rate from other questionnaire surveys in Poland. For instance the overall survey participation rate was 60% in the GATS Poland but in the Multi-Centre National Population Health Examination Survey (WOBASZ II study) the response rate was 45.5% [21,31,32]. The recipients of social care derived from the local government records and all of the people who met eligible criteria were invited to participate. The age, gender distribution of the respondents has been checked and we have not found a statistically significant difference of the age and gender structure in the group of the respondents compared to the non-respondents group. The third limitation of the study is related to the fact that our results cannot be generalized to all the Polish population of the disadvantaged citizens, as we only researched one region in Poland (Piotrkowski district within Lodzkie voivodship - rural disadvantaged area). In addition, there was no objective validation of tobacco/ETS cigarette use. The data were based on self-report, which may be prone to response bias. However, the likelihood of response bias was assumed to be minimal since the survey was conducted so that the respondents remained anonymous. It also needs to be added that the study has evaluated different factors which might be correlated with the level of knowledge about harmful effect of smoking in order to identify important groups within this vulnerable population. However, these measures may not capture the full spectrum of the vulnerability. Finally, all the questions were asked with a negative focus. To ensure good internal validity it would be recommended to include a mixture of positive and negative statements for the participants to agree or disagree with, so as not to unintentionally direct the response.

## 5. Conclusions

The results of our study underline the need to improve knowledge on the dangers of active and passive smoking among the disadvantaged segments of the population. There is a need to focus on some specific diseases attributed to smoking and to be aware of the fact that vulnerable groups, including male and those with the worst socio-economic status lack this knowledge.

## Figures and Tables

**Table 1:** Lack of awareness of smoking-associated health risks among the study respondents *n* = 1817.

Variable	Awareness of Smoking-Associated Health Risks							
	*Smoking Causes Serious Illnesses*		*Smoking Causes Strokes*		*Smoking Causes Heart Attacks*		*Smoking Causes Lung Cancer*	
	Unadjusted	Adjusted	Unadjusted	Adjusted	Unadjusted	Adjusted	Unadjusted	Adjusted
	Odds Ratio (95% CI)							
Gender								
Men	1.79 (1.26–2.54) ***	1.47 (1.02–2.11) *	1.06 (0.86–1.29)		1.07 (0.86–1.32)		2.21 (1.57–3.11) ***	1.86 (1.29–2.68) ***
Women	1.00 Reference	1.00 Reference	1.00 Reference		1.00 Reference		1.00 Reference	1.00 Reference
Age (years)								
18–29	0.86 (0.39–1.89)		1.15 (0.78–1.68)		1.22 (0.82–1.82)		0.49 (0.24–1.01) *	
30–39	1.20 (0.67–2.16)		0.88 (0.65–1.20)		0.81 (0.59–1.12)		0.56 (0.34–0.92) *	
40–49	1.25 (0.69–2.29)		0.92 (0.68–1.26)		0.93 (0.67–1.29)		0.85 (0.52–1.40)	
50–59	1.00 Reference		1.00 Reference		1.00 Reference		1.00 Reference	
Education								
Primary	2.86 (0.87–9.41)		0.70 (0.45–1.08)		1.00(0.62–1.60)		5.90 (1.41–24.62) **	
Vocational	2.99 (0.91–9.77)		0.83 (0.53–1.27)		0.94 (0.59–1.50)		4.58 (1.10–19.11) *	
Secondary	2.17 (0.66–7.18)		0.92 (0.60–1.42)		0.97 (0.61–1.55)		2.89 (0.68–12.20)	
High	1.00 Reference		1.00 Reference		1.00 Reference		1.00 Reference	
Employment status								
Permanent job	1.00 Reference		1.00 Reference		1.00 Reference		1.00 Reference	1.00 Reference
Temporary job	1.63 (0.90–2.99)		1.12 (0.78–1.60)		1.27 (0.87–1.27)		2.63 (1.44–4.79) **	2.14 (1.16–3.96) **
Disabled or retired	1.07 (0.37–3.12)		0.83 (0.46–1.51)		0.95 (0.50–1.82)		2.66 (1.11–6.40) *	2.32 (0.95–5.68)
Unemployed	1.06 (0.71–1.59)		0.96 (0.78–1.18)		1.08 (0.86–1.36)		1.62 (1.05–2.50) *	1.66 (1.07–2.59) *
Subjective assessment of monthly income								
Sufficient to cover all living needs plus may save a certain amount	1.21 (0.27–5.43)		1.32 (0.53–3.24)	1.32 (0.53–3.24)	1.63 (0.65–4.10)	1.63 (0.65–4.10)	–	
Sufficient to cover all living needs	0.65 (0.32–1.29)		1.10 (0.78–1.55)	1.10 (0.78–1.55)	0.94 (0.64–1.37)	0.94 (0.64–1.37)	0.53 (0.27–1.05)	
Sufficient to cover basic needs only	0.86 (0.57–1.30)		1.14 (0.90–1.44)	1.14 (0.90–1.44)	1.05- (0.82–1.35)	1.05 (0.82–1.35)	0.68 (0.46–1.00) *	
Declined response or difficult to say	1.05 (0.58–1.89)		1.81 (1.29–2.54) ***	1.81 (1.29–2.54) ***	1.43 (1.00–2.03) *	1.43 (1.00–2.03) *	1.03 (0.60–1.78)	
Not sufficient to cover even the basic needs	1.00 Reference		1.00 Reference	1.00 Reference	1.00 Reference	1.00 Reference	1.00 Reference	
Smoking status								
Smokers	2.56 (1.80–3.65)	2.35 (1.63–3.38) ***	1.05 (0.87–1.28)		1.09 (0.88–1.34)		2.82 (1.99–3.99) **	2.31 (1.60–3.32) ***
Non-smokers	1.00 Reference	1.00 Reference	1.00 Reference		1.00 Reference		1.00 Reference	1.00 Reference

* *p* < 0.05; ** *p* < 0.01; *** *p* < 0.001.

**Table 2:** Lack of awareness of environmental tobacco smoke exposure-associated health risks among the study respondents *n* = 1817.

Variable	Awareness of Environmental Tobacco Smoke Exposure-Associated Health Risks							
	*ETS Causes Serious Illnesses*		*ETS Causes Strokes*		*ETS Causes Heart Attacks*		*ETS Causes Lung Cancer*	
	Unadjusted	Adjusted	Unadjusted	Adjusted	Unadjusted	Adjusted	Unadjusted	Adjusted
	Odds Ratio (95% CI)							
Gender								
Men	1.13 (0.91–1.40)		1.09 (0.89–1.33)		1.17 (0.95–1.45)		0.95 (0.71–1.28)	
Women	1.00 Reference		1.00 Reference		1.00 Reference		1.00 Reference	
Age (years)								
18–29	1.47 (0.97–2.23)		1.02 (0.70–1.49)		0.92 (0.61–1.38)		1.34 (0.74–2.41)	
30–39	1.31 (0.94–1.82)		0.92 (0.69–1.25)		0.96 (0.70–1.31)		1.54 (0.96–2.47)	
40–49	1.22 (0.87–1.72)		0.96 (0.71–1.31)		1.06 (0.77–1.48)		1.22 (0.74–2.01)	
50–59	1.00 Reference		1.00 Reference		1.00 Reference		1.00 Reference	
Education								
Primary	0.76 (0.48–1.21)		1.26 (0.80–1.98)		1.00 (0.80–1.25)		0.99 (0.51–1.92)	
Vocational	0.70 (0.44–1.11)		1.09 (0.70–1.70)		1.02 (0.75–1.38)		0.95 (0.49–1.83)	
Secondary	0.87 (0.55–1.37)		1.13 (0.72–1.76)		0.87 (0.64–1.18)		1.19 (0.62–2.27)	
High	1.00 Reference		1.00 Reference		1.00 Reference		1.00 Reference	
Employment status								
Permanent job	1.00 Reference		1.00 Reference	1.00 Reference	1.00 Reference		1.00 Reference	
Temporary job	1.20 (0.82–1.77)		1.52 (1.06–2.18) *	1.52 (1.06–2.18) *	1.23 (0.84–1.79)		0.80 (0.46–1.38)	
Disabled or retired	1.71 (0.96–3.03)		1.02 (0.57–1.85)	1.02 (0.57–1.85)	0.75 (0.39–1.43)		0.49 (0.17–1.39)	
Unemployed	1.08 (0.86–1.35)		1.51 (1.22–1.87) ***	1.51 (1.22–1.87) ***	1.11 (0.89–1.39)		0.91 (0.67–1.23)	
Subjective assessment of monthly income								
Sufficient to cover all living needs plus may save a certain amount	1.32 (0.53–3.31)	1.32 (0.53–3.31)			0.63 (0.23–1.78)	0.63 (0.23–1.78)	1.18 (0.34–4.16)	
Sufficient to cover all living needs	0.86 (0.60–1.23)	0.86 (0.60–1.23)			0.79 (0.55–1.14)	0.79 (0.55–1.14)	1.16 (0.72–1.87)	
Sufficient to cover basic needs only	0.71 (0.56–0.90) **	0.71 (0.56–0.90) **			0.76 (0.60–0.97) *	0.76 (0.60–0.97) *	1.05 (0.75–1.46)	
Declined response or difficult to say	1.35 (0.96–1.89)	1.35 (0.96–1.89)			1.20 (0.85–1.69)	1.20 (0.85–1.69)	0.68 (0.40–1.18)	
Not sufficient to cover even the basic needs	1.00 Reference	1.00 Reference	1.00 Reference		1.00 Reference	1.00 Reference	1.00 Reference	
Smoking status								
Smokers	1.02 (0.83–1.26)		0.99 (0.81–1.20)		1.15 (0.94–1.41)		0.85 (0.64–1.13)	
Non-smokers	1.00 Reference		1.00 Reference		1.00 Reference		1.00 Reference	

* *p* < 0.05; ** *p* < 0.01; *** *p* < 0.001.

**Table 3:** Awareness Index (AI) in relation to the number of cigarettes smoked daily, number of previous quitting attempts and intention to quit smoking within the next month.

Variable	Smoking Causes Serious Illness		*Smoking Causes Strokes*		*Smoking Causes Heart Attacks*		*Smoking Causes Lung Cancer*		Awareness Index (AI)	
	Aware	Not Aware	Aware	Not Aware	Aware	Not Aware	Aware	Not Aware	AI 0–3	AI 4
Number of cigarettes smoked daily Mean (±SD)	12.84 ± 7.70	14.65 ± 7.37	12.94 ± 7.84	13.31 ± 7.54	12.82 ± 7.64	13.70 ± 7.85	12.92 ± 7.68	13.92 ± 7.68	13.27 ± 7.76	12.95 ± 7.70
Intention to quit smoking within the next month	105 (17.4)	7 (9.3)	76 (19.3)	35 (13.0)	82 (17.5)	29 (14.5)	7 (8.4)	7 (8.4)	39 (13.1)	72 (19.7) *
Number of previous quit attempts Mean (±SD)	3.13 ± 11.22	5.33 ± 18.61	3.87 ± 13.99	2.81 ± 9.98	3.55 ± 12.79	3.12 ± 11.61	3.64 ± 13.20	1.72 ± 0.45	2.73 ± 9.57	4.02 ± 14.47

* *p* < 0.05.

## Supplementary Materials

The following are available online at http://www.mdpi.com/1660-4601/15/10/2244/s1, Table S1: Characteristics of the study respondents.
