# Supplementary material for: Tobacco Health Risk Awareness among Socially Disadvantaged People—A Crucial Tool for Smoking Cessation
Source: Int J Environ Res Public Health. 2018 Oct 13;15(10):2244. doi: 10.3390/ijerph15102244 (PMC6211097; doi:10.3390/ijerph15102244)
Supplement: Supplementary file 1 [file ijerph-15-02244-s001.pdf]

Supplementary Information

Tobacco Health Risk Awareness among Socially Disadvantaged People—A Crucial Tool for Smoking Cessation

Marek Milcarz [1], Kinga Polańska [1,*], Leokadia Bak-Romaniszyn [2] and Dorota Kaleta [1]

1 Department of Hygiene and Epidemiology, Medical University of Lodz, 90-647 Lodz, Poland; marek.milcarz@op.pl (M.M.); dkaleta@op.pl (D.K.)
2 Department of Nutrition in Digestive Tract Diseases, Medical University of Lodz, 90-647 Lodz, Poland; leokadia.bak-romaniszyn@umed.lodz.pl
* Correspondence: kinga.polanska@umed.lodz.pl; Tel.: +48-604-397-242

Table S1. Characteristics of study respondents n = 1817.

Variable	Total Sample	Women				Men			
		Total	Smokers	Non-Smokers		Total	Smokers	Non-Smokers	
**Overall**	***n* = 1817**	***n* = 1224**	***n* = 362 (29.6%)**	***n* = 862 (70.4%)**	*p*	***n* = 593**	***n* = 313 (52.8%)**	***n* = 280 (47.2%)**	*p*
	***n* (%)**	***n* (%)**	***n* (%)**	*n* (%)		***n* (%)**	***n* (%)**	***n* (%)**	
**Age (years)**									
18–29	209 (11.5)	160 (76.6)	45 (28.1)	115 (71.9)		49 (23.4)	30 (61.2)	19 (38.8)	
30–39	778 (42.8)	566 (72.8)	151 (26.7)	415 (73.3)	$p > 0.05$	212 (27.2)	102 (48.1)	110 (51.9)	$p > 0.05$
40–49	601 (33.1)	385 (64.1)	129 (33.5)	256 (66.5)		216 (35.9)	111 (51.4)	105 (48.6)	
50–59	229 (12.6)	113 (49.3)	37 (32.7)	76 (67.3)		116 (50.7)	70 (60.3)	46 (39.7)	
**Education**									
Primary	493 (27.7)	283 (57.4)	113 (39.9)	170 (60.1)		210 (42.6)	137 (65.2)	73 (34.8)	
Vocational	586 (32.9)	351 (59.9)	113 (32.2)	238 (67.8)	$p < 0.0001$	235 (40.1)	128 (54.5)	107 (45.5)	$p < 0.0001$
Secondary	606 (34.0)	475 (78.4)	122 (25.7)	353 (74.3)		131 (21.6)	43 (32.8)	88 (67.2)	
High	96 (5.4)	88 (91.7)	7 (8.0)	81 (92.0)		8 (8.3)	1 (12.5)	7 (87.5)	
Missing data	36 (2.0)	27 (2.2)	7 (1.9)	20 (2.3)		9 (1.5)	4 (1.3)	5 (1.8)	
**Employment status**									
permanent job	533 (29.5)	310 (58.2)	79 (25.5)	231 (74.5)		223 (41.8)	82 (36.8)	141 (63.2)	
temporary job	156 (8.6)	85 (54.5)	34 (40.0)	51 (60.0)		71 (45.5)	47 (66.2)	24 (33.8)	
disabled or retired	54 (3.0)	26 (48.1)	6 (23.1)	20 (76.9)	$p > 0.05$	28 (51.9)	15 (53.6)	13 (46.4)	$p < 0.0001$
Student	2 (0.1)	2 (100.0)	1 (50.0)	1 (50.0)		0	0	0	
unemployed	1060 (58.7)	793 (74.8)	241 (30.4)	552 (69.6)		267 (25.2)	167 (62.5)	100 (37.5)	
Missing data	12 (0.7)	8 (0.7)	1 (0.3)	7 (0.8)		4 (0.7)	2 (0.6)	2 (0.7)	

Table S1. *Cont.*

**Subjective assessment of monthly income**									
sufficient to cover all living needs plus may save a certain amount	20 (1.1)	16 (80.0)	2 (12.5)	14 (87.5)		4 (20.0)	2 (50.0)	2 (50.0)	
sufficient to cover all living needs	198 (10.9)	143 (72.2)	26 (18.2)	117 (81.8)		55 (27.8)	14 (25.5)	41 (74.5)	
sufficient to cover basic needs only	933 (51.5)	650 (69.7)	193 (29.7)	457 (70.3)	$p < 0.0001$	283 (30.3)	142 (50.2)	141 (49.8)	$p < 0.0001$
not sufficient to cover even the basic needs	454 (25.1)	266 (58.6)	101 (38.0)	165 (62.0)		188 (41.4)	125 (66.5)	63 (33.5)	
Declined response	64 (3.5)	42 (65.6)	7 (16.7)	35 (83.3)		22 (34.4)	8 (36.4)	14 (63.6)	
difficult to say	141 (7.8)	101 (71.6)	32 (31.7)	69 (68.3)		40 (28.4)	21 (52.5)	19 (47.5)	
Missing data	7 (0.4)	6 (0.5)	1 (0.3)	5 (0.6)		1 (0.2)	1 (0.3)	0 (0.0)	
**Subjective health state**									
Fair	632 (35.0)	434 (35.6)	112 (25.8)	322 (74.2)		198 (34.3)	107 (54.0)	91 (46.0)	
rather fair	559 (31.0)	405 (33.6)	121 (29.9)	284 (60.1)		154 (26.2)	77 (50.0)	77 (50.0)	
neither fair nor poor	420 (23.3)	275 (22.6)	89 (32.4)	186 (67.6)	$p > 0.05$	145 (24.7)	78 (53.8)	67 (46.2)	$p > 0.05$
rather poor	141 (7.8)	80 (6.6)	31 (38.7)	49 (61.3)		61 (10.4)	30 (49.2)	31 (50.8)	
Poor	53 (2.9)	24 (2.0)	7 (29.2)	17 (60.8)		29 (4.9)	20 (69.0)	9 (31.0)	
Missing data	12 (0.7)	6 (0.5)	2 (0.6)	4 (0.5)		6 (1.0)	1 (0.3)	5 (1.8)	
**Number of health problems**									
None	245 (13.7)	137 (11.4)	35 (25.5)	102 (74.5)		108 (18.4)	61 (56.5)	47 (43.5)	
1–3 health problems	968 (54.3)	645 (53.8)	176 (27.3)	469 (72.7)	$p > 0.05$	323 (55.1)	176 (54.5)	147 (45.5)	$p > 0.05$
4–6 health problems	469 (26.3)	344 (28.7)	115 (33.4)	229 (66.6)		125 (21.3)	58 (46.4)	67 (53.6)	
≥ 7 health problems	102 (5.7)	72 (6.0)	24 (33.3)	48 (66.7)		30 (5.1)	13 (43.3)	17 (56.7)	
Missing data	33 (1.8)	26 (2.1)	12 (2.1)	14 (1.6)		7 (1.2)	5 (1.6)	2 (0.7)	
**Awareness of smoking-associated health risks**									
Yes	1677 (92.4)	1147 (93.9)	320 (27.9)	827 (72.1)	$p < 0.0001$	530 (89.5)	274 (51.7)	256 (48.3)	$p > 0.05$
No	137 (7.6)	75 (6.1)	41 (54.7)	34 (35.3)		62 (10.5)	39 (62.9)	23 (37.1)	
Missing data	3 (0.2)	2 (0.2)	1 (0.3)	1 (0.1)		1 (0.2)	0 (0.0)	1 (0.4)	
**Awareness of smoking-associated health risks-stroke**									
Yes	1034 (56.9)	701 (57.3)	207 (29.5)	494 (70.5)		333 (56.2)	172 (51.6)	161 (48.4)	
No	748 (41.2)	498 (40.7)	147 (29.5)	351 (70.5)	$p > 0.05$	250 (42.1)	136 (54.4)	114 (45.6)	$p > 0.05$
Missing data	35 (1.9)	25 (2.0)	8 (2.2)	17 (2.0)		10 (1.7)	5 (1.6)	5 (1.8)	
**Awareness of smoking-associated health risks-heart attack**									
Yes	1244 (68.5)	843 (68.9)	248 (29.4)	595 (70.6)		401 (67.6)	207 (51.6)	194 (48.4)	
No	543 (29.9)	360 (29.4)	107 (29.7)	253 (70.3)	$p > 0.05$	183 (30.9)	102 (55.7)	81 (44.3)	$p > 0.05$
Missing data	30 (1.6)	21 (1.7)	7 (1.9)	14 (1.6)		9 (1.5)	4 (1.3)	5 (1.8)	
**Awareness of smoking-associated health risks-lung cancer**									
Yes	1672 (92.0)	1151 (94.0)	318 (27.6)	833 (62.4)		521 (87.9)	269 (51.6)	252(48.3)	
No	144 (7.9)	72 (5.9)	43 (59.7)	29 (40.3)	$p < 0.0001$	72 (12.1)	44 (61.1)	28 (38.9)	$p > 0.05$
Missing data	1 (0.1)	1 (0.1)	1 (0.3)	0 (0.0)		0 (0.0)	0 (0.0)	0 (0.0)	
**Awareness of ETS exposure-associated health risks**									
Yes	1258 (69.4)	858 (70.1)	259 (30.2)	599 (69.8)		400 (67.4)	208 (52.0)	192 (48.0)	
No	548 (30.2)	359 (29.3)	102 (28.4)	257 (71.6)	$p > 0.05$	189 (39.9)	104 (55.0)	85 (45.0)	$p > 0.05$
Missing data	11 (0.6)	7 (0.6)	1 (0.3)	6 (0.7)		4 (0.7)	1 (0.3)	3 (1.1)	

Table S1. *Cont.*

**Awareness of ETS exposure-associated health risks-stroke**									
Yes	1030 (56.7)	702 (57.3)	211 (30.1)	491 (69.9)		328 (55.3)	173 (52.7)	155 (47.3)	
No	769 (42.3)	510 (41.7)	148 (29.0)	362 (71.0)	$p > 0.05$	259 (43.7)	136 (52.5)	123 (47.5)	$p > 0.05$
Missing data	18 (1.0)	12 (1.0)	3 (0.8)	9 1.0)		6 (1.0)	4 (1.3)	2 (07)	
**Awareness of ETS exposure -associated health risks-heart attack**									
Yes	1243 (68.4)	850 (69.4)	249 (29.3)	601 (70.7)		393 (66.3)	200 (50.9)	193 (49.1)	
No	566 (31.2)	367 (30.0)	111 (30.2)	256 (69.8)	$p > 0.05$	199 (33.6)	112 (56.3)	87 (43.7)	$p > 0.05$
Missing data	8 (0.4)	7 (06)	2 (0.6)	5 (0.6)		1 (02)	1 (0.3)	0 (0.0)	
**Awareness of ETS exposure -associated health risks-lung cancer**									
Yes	1579 (86.9)	1061 (86.7)	320 (30.2)	741 (69.8)		518 (87.3)	275 (53.1)	243 (46.9)	
No	236 (13.0)	161 (13.1)	42 (26.1)	119 (73.9)	$p > 0.05$	75 (12.7)	38 (50.7)	37 (49.3)	$p > 0.05$
Missing data	2 (0.1)	2 (0.2)	0 (00)	2 (0.2)		0 (0.0)	0 (0.0)	0 (0.0)	
**ETS exposure (total number of hours per day**									
0	1041 (57.6)	744 (61.1)	149 (20.0)	595 (80.0)		297 (50.2)	100 (33.7)	197 (66.3)	
<1h	390 (21.6)	268 (22.0)	109 (40.7)	159 (59.3)		122 (20.4)	70 (57.4)	52 (42.6)	
1–5h	162 (9.0)	90 (7.4)	32 (35.6)	58 (64.4)	$p < 0.0001$	72 (12.2)	55 (76.4)	17 (23.6)	$p < 0.0001$
5–8h	76 (4.2)	44 (3.6)	21 (47.7)	23 (52.3)		32 (5.4)	22 (68.8)	10 (31.3)	
More than 8h	140 (7.7)	72 (5.9)	48 (66.7)	24 (33.3)		68 (11.5)	64 (94.1)	4 (5.9)	
Missing data	8 (0.4)	6 (0.5)	3 (0.8)	3 (0.3)		2 (0.3)	2 (0.6)	0 (0.0)	

ETS: Environmental Tobacco Smoke.
